# Nano-Biocomposite Materials Obtained from Laser Ablation of Hemp Stalks for Medical Applications and Potential Component in New Solar Cells

**DOI:** 10.3390/ijms24043892

**Published:** 2023-02-15

**Authors:** Alexandru Cocean, Georgiana Cocean, Maria Diaconu, Silvia Garofalide, Francisca Husanu, Bogdanel Silvestru Munteanu, Nicanor Cimpoesu, Iuliana Motrescu, Ioan Puiu, Cristina Postolachi, Iuliana Cocean, Silviu Gurlui

**Affiliations:** 1 Atmosphere Optics, Spectroscopy and Laser Laboratory (LOASL), Faculty of Physics, Alexandru Ioan Cuza University of Iasi, 11 Carol I Bld., 700506 Iasi, Romania; 2 Laboratory of Applied Meteorology and Climatology, RECENT AIR, Research Center with Integrated Techniques for Atmospheric Aerosol Investigation in Romania, Alexandru Ioan Cuza University of Iasi, A Building, Physics, 11 Carol I, 700506 Iasi, Romania; 3 Rehabilitation Hospital Borsa, 1 Floare de Colt Street, 435200 Borsa, Romania; 4 Faculty of Material Science and Engineering, Gheorghe Asachi Technical University of Iasi, 59A Mangeron Bld., 700050 Iasi, Romania; 5 Sciences Department & Research Institute for Agriculture and Environment, Iasi University of Life Sciences, 3 Sadoveanu Alley, 700490 Iasi, Romania; 6 Plants Sciences Department, Faculty of Agriculture, Iasi University of Life Sciences, 3 Sadoveanu Alley, 700490 Iasi, Romania

**Keywords:** Cannabis Sativa, hemp stalk thin film, drug delivery devices, solar cells, enhanced calcium deposition from polymer matrix

## Abstract

The study in this paper presents a new material that was produced as a thin film by the Pulsed Laser Deposition technique (PLD) using a 532 nm wavelength and 150 mJ/pulse laser beam on the hemp stalk as target. The analyses performed by spectroscopic techniques (Fourier Transform Infrared Spectroscopy—FTIR, Laser—Induced Fluorescence Spectroscopy—LIF, Scanning Electron Microscopy coupled with Energy Dispersive X-ray—SEM-EDX, Atomic Force Microscopy—AFM and optical microscope) evidenced that a biocomposite consisting of lignin, cellulose, hemicellulose, waxes, sugars and phenolyc acids *p*-coumaric and ferulic, similar to the hemp stalk target was obtained. Nanostructures and aggregated nanostructures of 100 nm to 1.5 μm size were evidenced. Good mechanical strength and its adherence to the substrate were also noticed. It was noticed that the content in calcium and magnesium increased compared to that of the target from 1.5% to 2.2% and from 0.2% to 1.2%, respectively. The COMSOL numerical simulation provided information on the thermal conditions that explain phenomena and processes during laser ablation such as C-C pyrolisis and enhanced deposition of calcium in the lignin polymer matrix. The good gas and water sorption properties due to the free OH groups and to the microporous structure of the new biocomposite components recommends it for studies for functional applications in medicine for drug delivery devices, filters in dialysis and for gas and liquid sensors. Functional applications in solar cells windows are also possible due to the conjugated structures of the contained polymers.

## 1. Introduction

The complex composite structure of hemp stalk makes it of interest for studies in order to develop new technologies of fabrication designed to enlarge the number of products that can be manufactured from Cannabis Sativa. Hemp is a plant of great industrial potential and has been used at least since five thousand years ago, with archaeological discoveries being recently reported [1]. The structure and components of hemp stalk have been studied and analyzed regarding its chemical composition and technical characteristics [2,3,4,5]. Influences of the soil composition induce specific characteristics to plant growth and stimulant treatments have been purposed for the better yield production of seeds and fibers [6]. Nowadays, interest in hemp has been growing due to the benefits that the plant provides for the environment, agriculture and industry of all kinds (textile, construction, dyes, food, pharmaceutics, cosmetics, etc.). Properties that have been associated with the plant such as antibacterial, antioxidative and others are now being confirmed and explained based on the studies performed on hemp constituents [7,8,9]. Additionally, polymers that are contained in hemp stalk have been studied for applications in pharmaceutical and cosmetic products, such as cellulose and cellulose nanocrystals [10,11]. Lignin, through its antioxidant capacity, is of potential use in the treatment of obesity, diabetes, thrombosis, viral infections and cancer, and lignin nanoparticles’ photoprotective effects on skin have been proven [12,13]. The health beneficial effects of the *p*-coumaric and ferulic acids for skin protection and treatment have also been studied [13,14]. The application of their use in skin depigmentation and hypopigmentation procedures [15,16] have been indicated. Materials for technologies that can be obtained from hemp stalk components have been proposed by researchers, such as optically transparent wood for transparent solar cell windows and other optical transparent-with-haze materials [17], and lignin optical parameters have been studied [18]. Lignin has become an attractive material for studies because of its potential functionalization and application in optoelectronics and biosensors [19,20].

The purpose of the study presented in this paper is to investigate the behavior of the main polymeric constituents of hemp stalk in interaction with the high-power-pulsed laser beam and to obtain thin films. The study resulted in obtaining a new material of chemical composition, similar to that of hemp stalk using the pulsed laser deposition (PLD) technique. As per our knowledge, this is the first study performed on hemp to obtain thin layers by laser ablation and deposition.

## 2. Results and Discussion

### 2.1. Micrography and Elemental Composition

**Figure 1:**
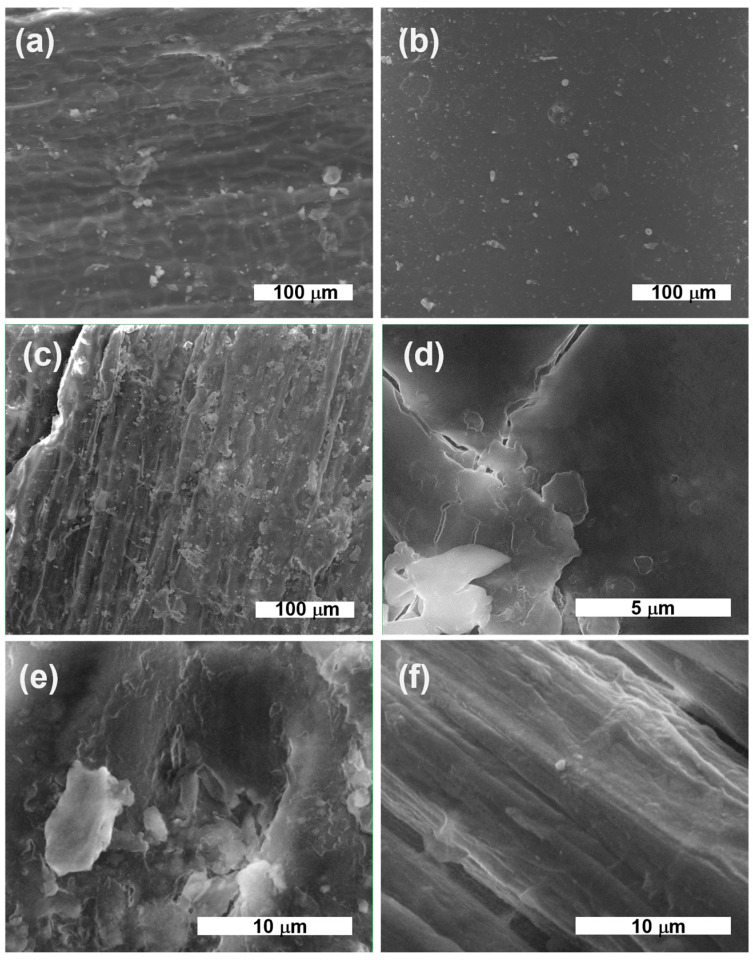
SEM images: HMP-STK target at deposition time (**a**) and the same HMP-STK target two years later (**c**,**e**); PLD-HMP-STK/Glass in 2020 (**b**) and in 2022 (**d**); thin film PLD-HMP-STK/HMP-FB as of 2022 (**f**).

**Figure 2:**
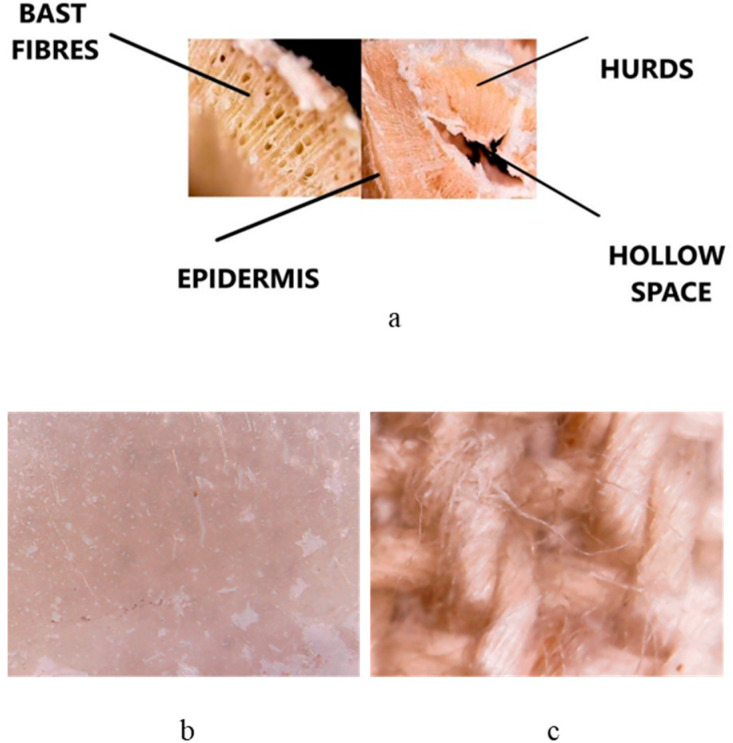
Images with an optical microscope of the HMP-STK cross-section (**a**), a thin film deposited on glass slab PLD-HMP-STK/Glass (**b**) and a thin film deposited on hemp fabric PLD-HMP-STK/HMP-FB (**c**).

After deposition, the HMP-STK target and the thin film deposited on glass slab were analyzed with electron microscopy (SEM) and the images are presented in Figure 1a,b. It can be observed that a continuous thin film was obtained consisting of droplets or agglomerations. Agglomerations can be also observed in the SEM images of the HMP-STLK (Figure 1a,c,e) and on the surface of the PLD-HMP-STK/HMP-FB sample (Figure 1f). The red-brownish color of the material of the PLD-HMP-STK thin films is observed in the optical microscopic images in Figure 2c,d. In Figure 1c, the transparency of the PLD-HMP-STK/Glass thin film can be noticed, as well as opaque, whitish formations that look like splashes. Their appearance corresponds to similar formations in the hemp stalk in the optical microscopic images of Figure 2a,b. The SEM images obtained two years after deposition of the thin films exhibit cracks in the PLD-HMP-STK/Glass due to multiple manipulations and the scratching procedure for FTIR analysis. However, we consider that the thin layer has a good mechanical strength and adhesion to the glass substrate. The elemental composition of the PLD-HMP-STK on glass and on HMP-FB is similar to the target elemental composition and presents inhomogeneities for different areas when analyzed by EDX. An increase in calcium and magnesium concentration can be observed from an average of 1.5% calcium and 0.2% magnesium in the HMP-STK target to an average of 2.2% calcium and 1.2% magnesium in the PLD-HMP-STK thin films, while carbon content decreased from an average of 66% in HMP-STK target to 50% or less in the PLD-HMP-STK thin films. Due to the inhomogeneous disposition of the constituents, a conclusion cannot be drawn in this regard.

### 2.2. AFM: Atomic Force Microscopy Nanosurf Easy Scan 2, Liestal, Switzerland

**Figure 3:**
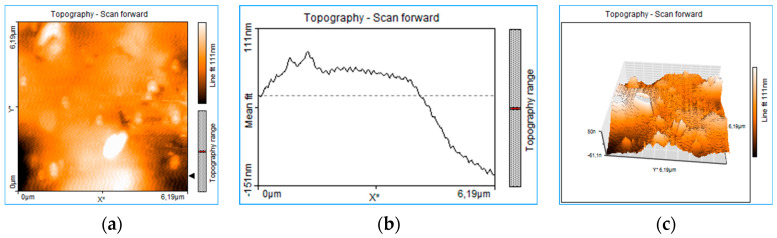

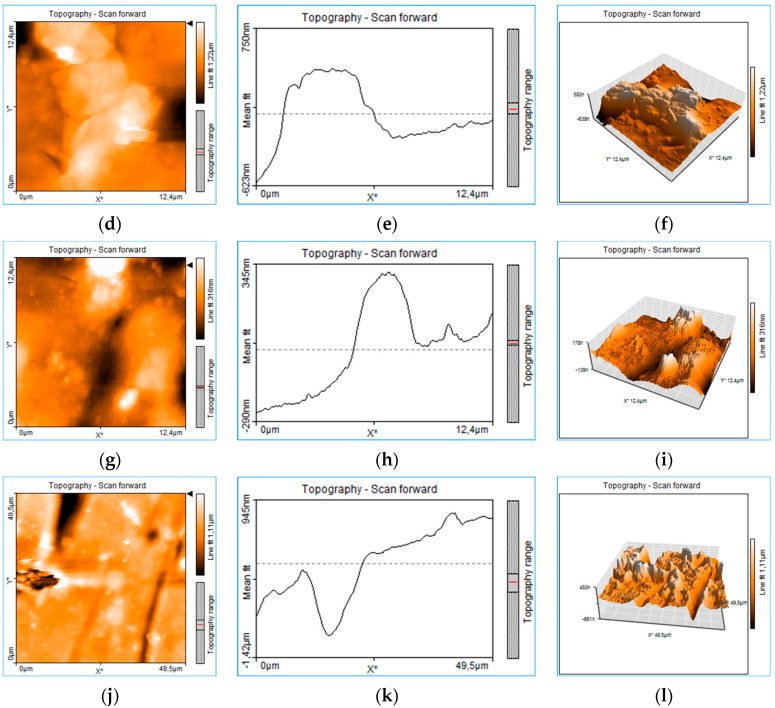
Roughness analysis of the PLD-HMP-STK thin film: 2D plots of surface topography (**a**,**d**,**g**,**j**), 1D plots of thickness topography (**b**,**e**,**h**,**k**) and 3D plots in volume topography (**c**,**f**,**i**,**l**).

The rough structure of the thin layer deposited on the glass (PLD-HMP-STK/Glass) of an unevenly distributed topography can be seen in Figure 3. Craters of 151 nm and 290 nm are noticed in the 1D topography plots on thin film thickness in Figure 3b,e. Aggregated structures and droplets of different sizes from 100 nm to 1.5 μm or even 6 μm are noticed in the 2D images of topography (Figure 3a,d,g,j). In Figure 3, the 1D plots (e) and (h) of the thin film topography show two structures assigned to droplets. The measurements of size droplets were performed on the *x*-axis as FWHM (full width at half maximum) and on the *z*-axis. The droplets’ widths were 4.78 μm and 2.610 μm, respectively, while the droplets’ heights were 357 nm and 98 nm. The topographic lines (Figure 3b,e,h,k) also indicate overlapping or clumped droplets by the jagged appearance of the lines [21], this indicates that most of the micrometric structures are in fact the result of nanostructure aggregation. The roughness is well observed in the 3D plots (Figure 3c,f,i,l). The AFM images were acquired after various manipulation including collecting material from the PLD-HMP-STK thin film for the FTIR analysis. Therefore, the “channels” of 623 nm to 1.42 μm depth observed in the plots of Figure 3g–l are due to cracking or they resulted during scratching for the sample collecting purpose.

### 2.3. Functional Groups Analysis in FTIR Spectroscopy

**Figure 4:**
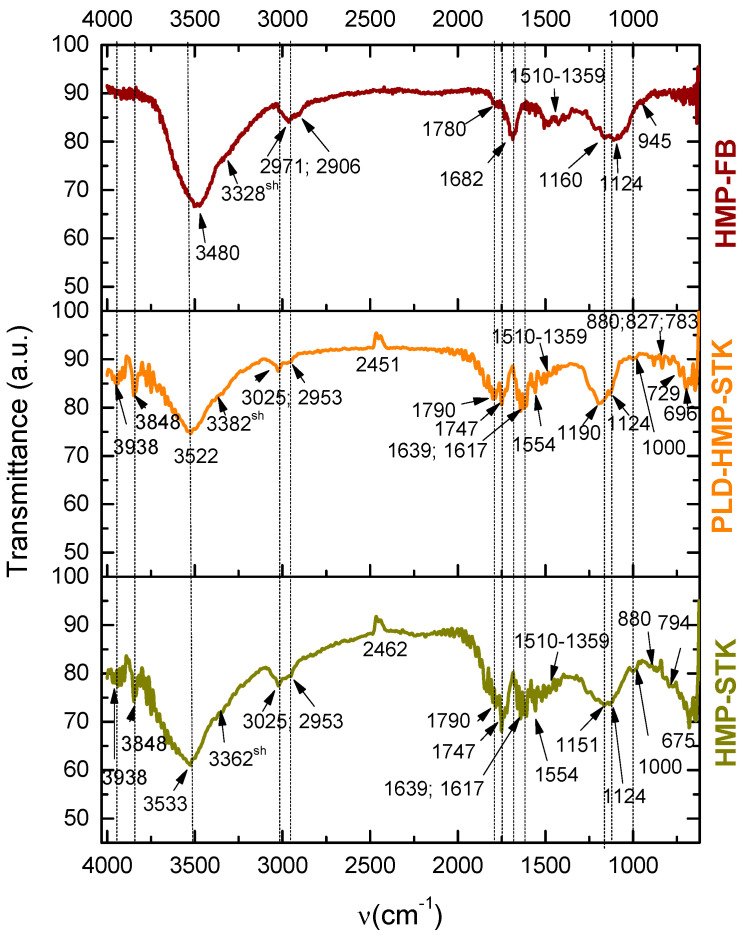
Fourier Transform Infrared Spectroscopy spectra: hemp stalk (HMP-STK); hemp fiber produced under the classical process for wet spinning (HMP-FB); thin film (PLD-HMP-STK) obtained under the PLD method applied to hemp stalk.

The FTIR spectra of the target HMP-STK and the thin film PLD-HMP-STK (Figure 4) are very similar, denoting that the functional groups are well preserved during laser ablation. The vibrational modes and corresponding functional groups are presented in Table 1. The bands in HMP-STK and PLD-HMP-STK spectra at 3938/3938 cm^−1^ and 3533/3522 cm^−1^ of O-H groups free and intermolecular bonded in polymers, 3025/3025 cm^−1^ of aryl groups, 1510/1510 cm^−1^ of C=C aromatic, 1359/1359 cm^−1^ of C-H and O-H bendings, as well as 675/696 cm^−1^ of O-H bendings are assigned to phenols in lignin and phenolic acids of parenchyma origin (*p*-coumaric acid and ferulic acid). The carboxyl groups in the *p*-coumaric and ferulic acids are evidenced by the 1790/1790 cm^−1^ and 1747/1747 cm^−1^ bands.

Hemp fabric (HMP-FB) made of yarns produced from hemp fiber from water-retted stalks was used to compare and to evidence the good preservation of the hemp stalk constituents in the PLD-HMP-STK. The HMP-FB spectrum was used to compare the results obtained by laser ablation and deposition. During the technological process for HMP-FB fabrication, the stalk was water-retted and the hurds (the wood in the HMP-STK) were mostly removed during the primary processing in the retting mills. After that, during preparation for spinning and weaving, the fiber lost more of the remaining wood and other components were removed through physical and chemical processes of combing, boiling, alkali treatments and bleaching with oxygen peroxide. The 2971 cm^−1^ peaks assigned by Malgorzata Zimniewska et al., 2018 [7] to aryl C-H in lignin combined with the 1510 cm^−1^ band of C=C aromatic symmetrical stretching vibrations [5,7,24] and the methoxyphenolic substitution in the aromatic ring denoted by the 1359 cm^−1^ peak also assigned to O-H bending in phenols and in alcohols [5,24], as well as the O-H vibrations in phenol polymers at 3480 cm^−1^, indicate that lignin is still present in the HMP-FB material. Additionally, a number of low intensity peaks in the 4000–3800 cm^−1^ range are noticed which can be assigned to free O-H stretching. The low intensity bands in the 650–700 cm^−1^ range can be assigned to C–H aromatic bending-out-of-plane modes. In the case of HMP-FB, the lignin noticed in the FTIR spectrum is contained in the fibers while the lignin evidenced in the PLD-HMP-STK is sourced in all of the hemp stalks’ structural constituents (wood, fibers etc.). Most of the phenolic peaks of the PLD-HMP-STK spectrum are identical to the HMP-STK spectrum, and only a slight shift in the C–H aromatic bending-out-of-plane mode is noticed from 675 cm^−1^ to 696 cm^−1^.

Cellulose is evidenced in all three spectra of HMP-STK, PLD-HMP-STK and HMP-FB by the peaks at 3362/3382/3328 cm^−1^ of O-H intermolecularly bonded in alcohol in polymers. The spectra of HMP-STK and PLD-HMP-STK also exhibit vibrations specific to free O-H in alcohols at 3848 cm^−1^, which are of very low intensity, with most missing in the HMP-FB spectrum. Alkyl groups of cellulose are denoted by the 2953/2953/2906 cm^−1^ peaks in the HMP-STK, PLD-HMP-STK and HMP-FB spectra known to be typical to carbohydrates, and the O-H bendings are indicated by the 1359/1359/1359 cm^−1^, 729/729/729 cm^−1^ peaks. Skeletal vibrations due to C-O-C in cyclic ethers are indicated in all three spectra by the peaks from 1151 cm^−1^ to 880 cm^−1^ wavenumbers. The characteristic vibrations in cellulose overlap with the characteristic vibrations in hemicellulose, starch, sugar and pectin.

**Figure 5:**
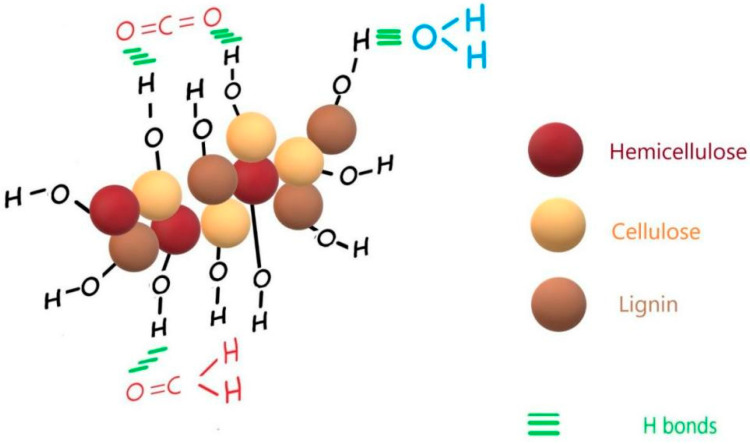
Schematical representation of CO_2,_ water and formaldehyde adsorption on PLD-HMP-STK thin film.

The adsorbed formaldehyde exhibited by the vibrations at 1554 cm^−1^ and 1747 cm^−1^ in HMP-STK and PLD-HMP-STK and which are missing in HMP-FB denote good adsorbing properties of the PLD-HMP-STK material. Adsorbed water and adsorbed carbon dioxide are also evidenced in the HMP-STK and PLD-HMP-STK spectra denoted by the peaks at 3938 cm^−1^, 3848 cm^−1^, and mainly by the peak at 1639 cm^−1^ for water and the peak at 2462 cm^−1^. These peaks are missing in the HMP-FB spectrum. These adsorbing properties are due to an effective microporous structure that can be noticed in the AFM images of the thin film of PLD-HMP-STK topography (Figure 3). Additionally, the free O-H phenolic and alcoholic groups in lignin and cellulose, respectively, indicated by the vibrations at 3938 cm^−1^ and 3848 cm^−1^, are available to H-bonding with the C=O groups in CO_2_ and in formaldehyde and with the O-H groups in water, as schematically represented in Figure 5.

Sorbent materials are of high interest in medicine but also in sensor applications. Recently, De Pascale, M et al., 2022 [30] published a study on cellulose acetate-based membranes for the removal of uremic toxins from dialysate. The adsorbing property of the polymeric thin film PLD-HMP-STK makes it a candidate to be used as a matrix in drug delivery devices, including transdermal patches. The components of PLD-HMP-STK thin film consisting of lignin, cellulose and, most importantly, phenolic carboxylic acids *p*-coumarin and ferulic, due to their antioxidant properties, are also recommended as an active material for transdermal patches for cosmetics and dermatology. The gas-adsorbing property of PLD-HMP-STK makes it a candidate material for gas sensing in medicine and for applications in other fields where gas sensing is required. Additionally, PLD-based techniques can be applied in 3D printing and the ablated material from HMP-STK can be used as a filler or to develop components for drug delivery devices. The PLD-HMP-STK thin film is also a candidate for solar cell windows.

### 2.4. Numerical Simulation in COMSOL 5.6

**Figure 6:**
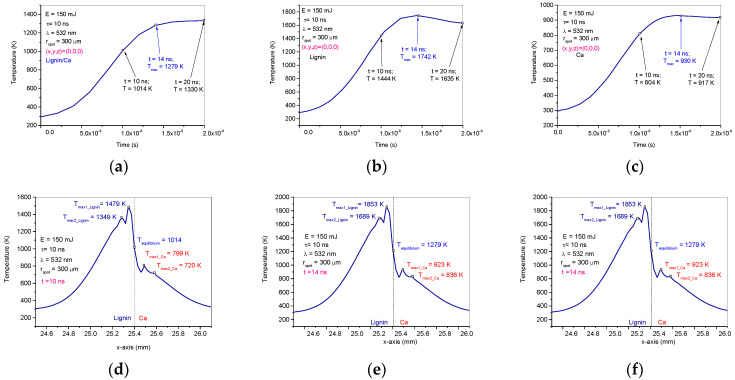
Pulsed laser heating effects on the lignin/Ca composite: Temperature diagrams in time on the spot center of the lignin/Ca composite target (**a**), of the calcium target (**b**) and of the lignin target (**c**); in pulse width on the lignin/Ca composite target (10 ns) (**d**), 14 ns after pulse laser ignition on the lignin/Ca composite target (**e**) and 20 ns after pulse laser ignition on the lignin/Ca composite target (**f**).

The diagrams in Figure 6 are 2D plots resulting from the simulation in COMSOL and show the temperature evolution during laser irradiation over time. The influences of heat diffusion between the composite lignin/Ca components are observed in Figure 6d–f where the maximum temperatures are shifted from the center of the spot (the center of the spot is at 25.4 mm in the diagrams) and where the maximum heating is obtained, which is usually when simulating the laser heating of homogeneous materials [21,31,32,33].

**Figure 7:**
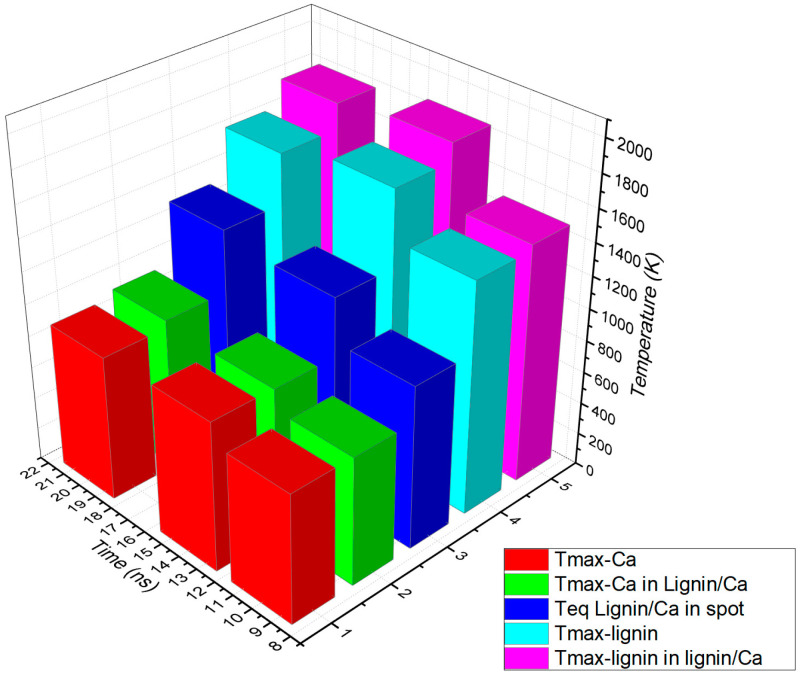
Plot of maximum temperatures achieved due to laser beam absorption and heat diffusion by calcium and lignin (Tmax-Ca and Tmax-lignin) compared to calcium and lignin as components in the lignin/Ca composite (Tmax-Ca in lignin/Ca and Tmax-lignin in lignin/Ca) and at an equilibrium in the spot center of the composite (Teq lignin/Ca in spot).

In order to evaluate the results of the simulation, the maximum temperatures in the individual targets of Ca and lignin and of each calcium and lignin component in the lignin/Ca composite as well as the temperatures of equilibrium were centralized in Table 2 based on the times when they were achieved. The data in Table 2 were plotted as shown in Figure 7. Compiling the optical and thermal parameters of the materials, the simulation shows enhancements of thermal effects in the lignin/Ca composite for both components (Tmax-Ca in lignin/Ca and Tmax-lignin in lignin/Ca) compared to the values achieved on the individual targets (Tmax-Ca and Tmax-lignin). Lignin better absorbs laser irradiation than calcium and laser heating is more effective on the component (Tmax-lignin in lignin/Ca) in the composite target. The maximum temperature of the component (Tmax-Ca in lignin/Ca) is considered the equilibrium temperature (Teq lignin/Ca in spot) achieved in the center of the spot due to laser heating and the thermal diffusion effect. 

The temperatures reached on the lignin component indicate conditions that can lead to processes similar to pyrolysis. Depolymerization of lignin is therefore expected in the sense that Kawamoto, H. et al., 2017 [34] describes lignin pyrolysis, thus meaning that C-C and C-O bonds attached to the aromatic ring may break. Under laser ablation conditions, recombinations of chemical structures resulting from the pyrolysis-like phenomena take place in the plasma of ablation. These recombinations are confirmed by the FTIR spectra. However, the temperatures achieved during laser ablation last only a few nanoseconds and could also contribute to the good preservation of the basic structure of lignin; lignin is only partially depolymerized, meaning that the result consists of a shorter polymer chain and does not lead to monomers.

The basic structures are not affected and comparing the FTIR spectra and SEM micrography of HMP-STK and PLD-HMP-STK (Figure 1 and Figure 4), as well as the AFM analyses performed on PLD-HMP-STK (Figure 3), there are indications that the material deposited in the PLD process consists of micrometric and submicrometric structures of the initial constituents of the hemp stalk biocomposite HMP-STK.

The simulation also provides information on the heating enhancement processes on both lignin and calcium; this could explain the increase in calcium content in the PLD-HMP-STK compared to the HMP-STK target.

### 2.5. LIF Spectroscopy Analysis

**Figure 8:**
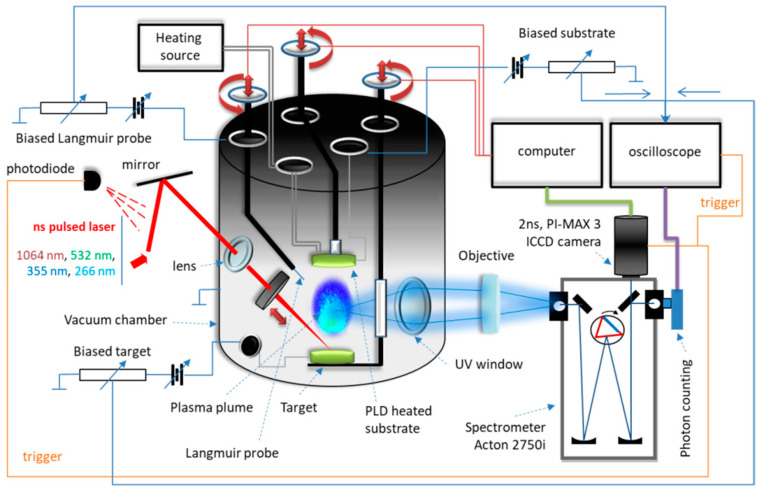
Experimental device.

**Figure 9:**
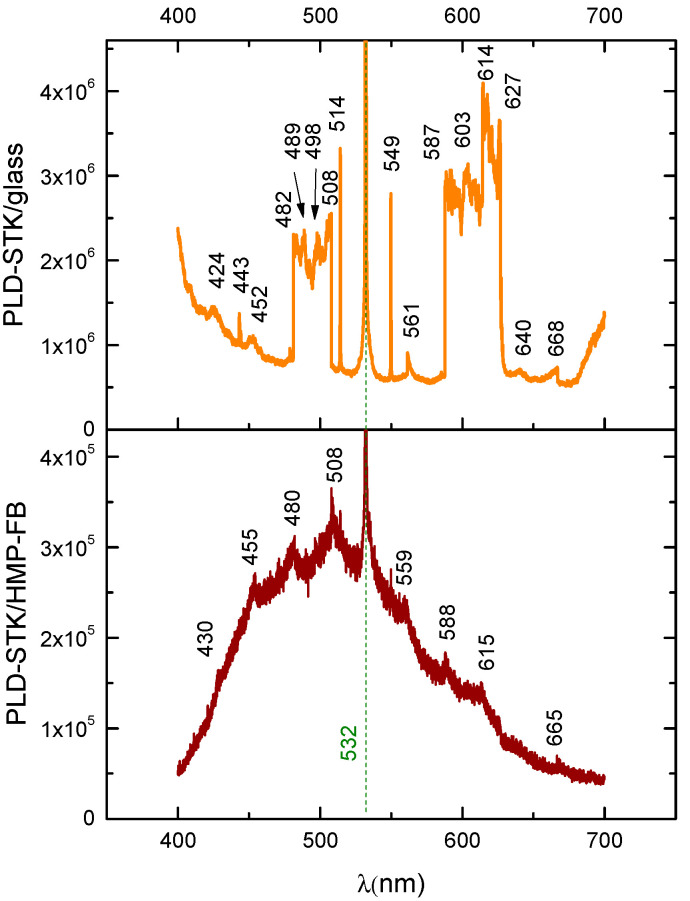
LIF spectra: thin film obtained by the PLD method using hemp stalk as a target deposited on different substrates: hemp fabric (PLD-STK/Hemp); glass slab (PLD-STK/glass).

For LIF spectroscopy performed on the thin films PLD-HMP-STK/Glass and PLD-HMP-STK/HMP-FB, an excitation laser beam of 355 nm was used, and the installation is presented in Figure 8. The characteristic fluorescence peak of the thin film PLD-HMP-STK/HMP-FB is noticed at 508 nm, as well as a number of secondary peaks due to chemical reactions [21,27,29] and to laser interactions with different constituents of the thin film, such as *p*-coumaric acid emitting at 430 nm and 455 nm [35,36] and ferulic acid with emissions noticed at 480 nm and 498 nm [37,38], as seen in the spectrum of Figure 9 and Table 3. In the LIF spectrum of PLD-HMP-STK/Glass (Figure 9 and Table 3), peaks of coumaric and ferulic acids are noticed at 424 nm, 443 nm, 452 nm, 482 nm and 498 nm, respectively, while peaks at 603 nm and 627 nm are assigned to chlorophyll [35,37,38]. Peaks at 549 nm, 561 nm, 587 nm, 603 nm, 614 nm, 640 nm and 668 nm denoting secondary reactions during 355 nm laser beam interactions with the PLD-HMP-STK/Glass thin film are also observed in the spectrum of Figure 9. The enhanced fluorescence intensity of PLD-HMP-STK/Glass compared to PLD-HMP-STK/HMP-FB can be explained due to the continuous phase of the former, while the latter thin film is more of a dispersion of nano- and microparticles within the fibers of the HMP-FB. The two tower bands in the ranges of 482–508 nm and 587–627 nm are assigned to a radical formation [29] and to depolymerization processes under laser irradiation.

Bathochromic and hypsochromic shifts are also noticed for similar peaks in the two spectra of Figure 9 and can be explained as an influence of the different deposition substrates, including their morphology, which affects the morphology of the film and its consistency.

## 3. Materials and Methods

### 3.1. Materials

The hemp stalk noted as HMP-STK (Figure 2a) used in the experiment is of Romanian provenience from the crop cultivated by SC Milen Tech SRL, the farm of Ripiceni, Botosani County, Romania in 2017. It is the Secuieni-Jubilee variety of monoic hemp created at S.C.D.A. Secuieni and approved in 2012.

### 3.2. Method of Work

After drying naturally at room temperature (20–25 °C) for one year, the hemp stalk was subject to different tests under laser irradiation. Two tests are presented in this paper, both using a YG 981E/IR-10 laser system: Quantel-YG980 Q-switched Nd:YAG laser, Quantel, Les Ulis, France. The installation is presented in Figure 8.

**Figure 10:**
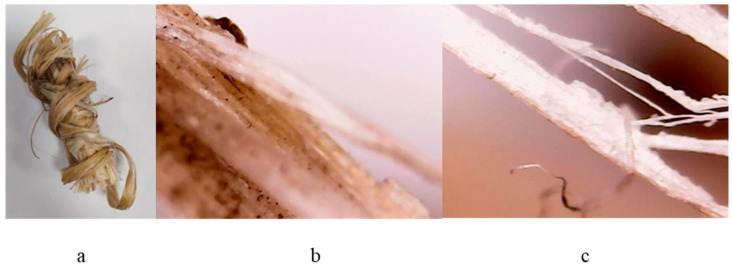
Crushed hemp stalk used as a target in the PLD process (**a**) and images with an optical microscope of the irradiated crushed hemp stalk (**b**,**c**).

The test was designed to obtain thin films by pulsed laser deposition (PLD), which involves controlled atmosphere laser ablation. Small bundles, as shown in Figure 10, were made from crushed stalk (simulating “decortication process”). The bundles resulted from the crushed hemp stalk (HMP-STK) were irradiated and the effects were studied while the method was improved until thin films were deposited on the glass slab (PLD-HMP-STK/Glass—Figure 2b) and on the hemp fabric (PLD-HMP-STK/HMP-FB—Figure 2c). 

The thin film deposition was conducted in the vacuum chamber of the laser installation. The 532 nm laser beam of 300 μm radius was used with a pulse width of 10 ns, 10 Hz repetition rate and 150 mJ/pulse. The pressure in the deposition chamber was of 10^−2^ Torr, the distance between the target to the support for thin film deposition was of 30 mm, and the deposition was 30 min long. The parameters and conditions for the PLD process were chosen in order to preserve most of the polymer chemical structure [21,27,29]. Additionally, the numerical simulation completed the information regarding plasma threshold conditions and for adjustments regarding the PLD procedure.

### 3.3. Methods of Analysis

#### 3.3.1. Numerical Simulation in COMSOL 5.6 Software

Numerical simulation was conducted in COMSOL 5.6 software version (COMSOL AB, Stockholm, Sweden) to evaluate the induced effects in a bi-component composite lignin/Ca. The optical parameters of lignin and calcium (refractive index *n* and extinction coefficient k) for laser irradiation used in the simulation were as per Alipoormazandarani et al., 2021 [18,19,20,39]. The theory and mathematics for the numerical model used were as per Cocean et al. [31,32,33]. 

#### 3.3.2. Fourier Transform Infrared Spectroscopy Analysis

The chemical composition was analyzed with Fourier Transform Infrared Spectroscopy Bomem MB154S spectrometer at an instrumental resolution of 4 cm^−1^ (Bomem, ABB group, Québec, QC, Canada). To prepare the pill for FTIR analysis, pieces of approximately 0.5 cm long were cut from the material used as a target; it was finally cut, then it was ground and mixed with potassium bromide in a mortar, then pressed into a pellet. For the thin layer (PLD-HMP-STK) analysis, the material was scraped from the surface of the film deposited on the glass slab and then mixed in a mortar together with KBr, after which the pellet was obtained with the hydraulic press [21,27,29].

#### 3.3.3. Laser-Induced Fluorescence Spectroscopy Analysis

The Laser-Induced Fluorescence (LIF) spectroscopy was performed using the 355 nm wavelength beam of the YG 981E/IR-10 laser system: Quantel-YG980 Q-switched Nd:YAG laser, Quantel, Les Ulis, France [27,29]. Both samples of thin films (PLD-HMP-STK/Glass and PLD-HMP-STK/HMP-FB) were used in LIF analysis.

#### 3.3.4. Scanning Electron Microscopy Coupled with Energy Dispersive X-ray Analysis

SEM-EDS was used to analyze the morphology and elemental composition: the Scanning Electron Microscope coupled with Energy Dispersive X-Ray (SEM-EDS) investigation with Vega Tescan LMH II, Brno, Cehia.

#### 3.3.5. Atomic Force Microscopy Analysis

The topography of PLD-HMP-STK/Glass was analyzed using AFM: Atomic Force Microscopy Nanosurf Easy Scan 2, Liestal, Switzerland.

## 4. Conclusions

The studies carried out for this paper led to obtaining a new material using the PLD technique. The new material consists of a thin film of nanostructures and aggregated nanostructures with chemical composition and composite constituents similar to that of the hemp stalk that was used as a target. These results, highlighted by spectral analysis, implicitly denote a good level of preservation of the functional groups and of the constituents of the hemp stalk biocomposite; this is better than in the case of the fabric obtained from hemp fibers manufactured by classical methods. The thin films show good mechanical strength and adhesion to the glass substrate.

The spectra of the thin films also show good water and gas sorption properties, which therefore recommend them as candidate materials for medical applications such as a matrix constituent in transdermal patches and in gas sensors. In addition, this type of thin film could be used for solar cell windows applications due to their components of conjugated chemical structures. 

The numerical simulation in COMSOL, providing information on temperatures achieved during ablation and temperature dynamics in time and space, contributed to a better and more extensive evaluation of the phenomena and processes that occurred during ablation and proved to be an important tool for performing and interpreting the experimental results.

## Figures and Tables

**Table 1:** Functional groups corresponding to the vibration bands in the spectra of Figure 4.

Vibration Bands [cm^−1^]			Functional Groups and References	Observations
HMP-STK	PLD-HMP-STK	HMP-FB		
3938	3938	-	O-H st free and bonded in phenols [22] O-H in adsorbed/absorbed water or in Mg-OH; Ca-OH [23]	lignin *p*-coumaric and ferulic acids (phenolic acids of parenchyma provenience) Mg-OH Ca-OH Adsorbed water
3848	3848	-	O-H st free in alcohols [24] O-H in carboxylic acid, known to be typical carbohydrates [25] O-H in adsorbed/absorbed water or in Mg-OH; Ca-OH [23,26]	cellulose, hemicellulose starch sugars Mg-OH Ca-OH adsorbed water
3533 3362	3522 3382	3480 3328	O-H st intermolecular bonded in alcohols and phenols in polymers [5,21] O-H st in carboxylic acids [24,27,28]	cellulose, hemicellulose, starch, sugars, lignin *p*-coumaric and ferulic acids
3025	3025	-	Aryl C-H groups [1,24]	lignin *p*-coumaric and ferulic acids
2953	2953	2971 2906	Alkyl C-H groups [8,21,24,26] 2971 cm^−1^-possible Aryl C-H in lignin [7]	cellulose, hemicellulose starch sugars Possible lignin
2462	2451	-	O=C=O st (carbon dioxide) [24,26]	adsorbed CO_2_
1790	1790	1780	C=O st in carboxyl groups in conjugated acids and aldehydes, esters [5,24,29] C=O st in vinyl/phenyl ester [5,24]	lignin pectin *p*-coumaric and ferulic acids waxes
1747	1747	-	C=O st in esters; [5,24] C=O st in carboxyl groups in conjugated acids of parenchyma and aldehydes, esters (in pectin, lignin, wax) [Ernö Pretch] $\mathrm{Adsorbed}\;\mathrm{formaldehyde}\;H_{2}C=O$ [24,26]	waxes *p*-coumaric and ferulic acids pectin lignin adsorbed formaldehyde
-	-	1682	C=O in carboxyl groups in carboxylic acids [8,24]; C=O in conjugated aldehide or ketone [24]	*p*-coumaric and ferulic acids
1639	1639	-	O-H of adsorbed water [5]; C=C st in monosubstituted alkene [24]	adsorbed water *p*-coumaric and ferulic acids
-	-	1617	C=C st in α,β-unsaturated ketone [24]	
1554	1554	-	$\mathrm{Adsorbed}\;\mathrm{formaldehyde}\;H_{2}C=O$ [24] C=O in carboxyl groups [4,21,24,26,28]	adsorbed formaldehyde *p*-coumaric and ferulic acids
1510	1510	1510	C=C aromatic symetrical st [4,5,24]	lignin
1359	1359	1359	C-H bending [24]; methoxyphenolic substitution in the aromatic ring [8,24]; H in-plane bending in phenols in lignin [24] O-H in-plane bending, intermolecular bonded in alcohols in polymers [5,24]	cellulose, hemicellulose starch sugars lignin
1151	1190	1160	Skeletal vibrations due to C-O-C asymmetric st in the oxane ring (cyclic ethers) [4,21,24]	cellulose (amorphous to crystalline 1160; 1190) Hemicellulose Starch pectin
1124	1124	1124	Skeletal vibrations due to C-O-C asymmetric st in the oxane ring (cyclic ethers) [21,24]	cellulose (amorphous to crystalline 1160; 1190) Hemicellulose Starch pectin
1000	1000	945	Skeletal vibrations due to C-O-C asymmetric st in the oxane ring (cyclic ethers) [24] Side groups vibrations [4,24] C=C bending in alkene in the carboxylic acids [24]	cellulose Hemicellulose Starch Pectin *p*-coumaric and ferulic acids
880	880	-	Skeletal vibrations due to C-O-C symmetric st, C-C-O and C-C-H bendings [4,5,21,24]; C=C bending in alkene in the carboxylic acids [24]	cellulose Hemicellulose Starch Pectin *p*-coumaric and ferulic acids
-	827	-	C=C bending in alkene trisubstituted [24]	*p*-coumaric and ferulic acids
794	783	-	Out-of-plane bending in alkene in the carboxylic acids [24]	*p*-coumaric and ferulic acids
729	729	729	CH_2_ rocking [24]; C=C bending in alkene disubstituted (cis) in the carboxylic acids [24] O-H out-of-plane bending [24] C-H bending [24]	Cellulose *p*-coumaric and ferulic acids
675	696	-	C-H bending and ring bending [24]; C=C bending in alkene disubstituted (cis) in the carboxylic acids [24] C–H aromatic bending-out-of-plane modes [26] Adsorbed molecular CO_2_ [26] O-H out-of-plane bending [24]; C-OH out-of-plane bending [5,24]	cellulose crystalline state, hemicellulose, starch, sugars, lignin *p*-coumaric and ferulic acids adsorbed CO_2_
650–624	650–624	650–624	O-H out-of-plane bending [24] C-OH out-of-plane bending [5,24]	cellulose, hemicellulose, starch, sugars, lignin *p*-coumaric and ferulic acids adsorbed water

**Table 2:** Compared maximum temperatures developed on Ca in the lignin/Ca composite at different times compared to temperatures in pure Ca at different times.

Time	10 ns	14 ns	20 ns
T_equilibrium lignin/Ca in spot center (x,y,z) = (0,0,0)_	1014 K	1279 K	1330 K
T_max-Ca (in lignin/Ca composite target)_	799 K 720 K	923 K 836 K	948 K 840 K
T_max-lignin (in lignin/Ca composite target)_	1479 K 1349 K	1853 K 1689 K	1792 K 1645 K
T_max-Ca in pure Ca target in spot center (x,y,z) = (0,0,0)_	804 K	930 K	904 K
T_max-lignin in pure lignin target in spot center (x,y,z) = (0,0,0)_	1444 K	1742 K	1635 K

**Table 3:** The fluorescence peaks of the thin films of PLD-HMP-STK/HMP-FB and PLD-HMP-STK/Glass.

Fluorecence [nm]		Emission, Fluorophores and References
PLD-HMP-STK/HMP-FB	PLD-HMP-STK/Glass	
430	424	Violet-blue due to *p*- coumaric acid and its derivatives [37,38]; bathochromic shift on PLD-HMP-STK/Glass
-	443	Blue due to coumaric acid derivatives [37,38]
455	452	Blue due to coumaric acid [37,38]; bathochromic shift on PLD-HMP-STK/Glass
480	482 v. strong	Blue-green due to ferulic acid [35,36]; slight hypsochromic shift
498	489 v. strong	Blue-green due to ferulic acid [35,36]; bathochromic shift and enhanced intensity due to the *p*-coumaric acid concentration [35] on PLD-HMP-STK/Glass
508 (max)	508 v. strong	Green; enhanced fluorescence intensity on PLD-HMP-STK/Glass
-	549	Green-yellow
559	561	Yellow-green; hypsochromic shift and enhanced fluorescence intensity on PLD-HMP-STK/Glass
588	587 v. strong	Yellow; slight hypsochromic shift and enhanced fluorescence intensity on PLD-HMP-STK/Glass
-	603 v. strong	Yellow-red; assigned to chlorophyll [35]
615	614 v. strong	Yellow-red; slight hypsochromic shift and enhanced fluorescence intensity on PLD-HMP-STK/Glass
-	627 v. strong	Red-yellow; assigned to chlorophyll [35]
-	640	Red-yellow
665	668	Red; slight hypsochromic shift and enhanced fluorescence intensity on PLD-HMP-STK/Glass

## Data Availability

Not applicable.

## References

[1] Singh M., Sardesai M.M. (2016). Cannabis sativa (Cannabaceae) in ancient clay plaster of Ellora Caves, India. Curr. Sci..

[2] Snegireva A., Chernova T., Ageeva M., Lev-Yadun S., Gorshkova T. (2015). Intrusive growth of primary and secondary phloem fibres in hemp stem determines fibre-bundle formation and structure. AoB PLANTS.

[3] Stevulova N., Cigasova J., Estokova A., Terpakova E., Geffert A., Kacik F., Singovszka E., Holub M. (2014). Properties Characterization of Chemically Modified Hemp Hurds. Materials.

[4] Zimniewska M. (2022). Hemp Fibre Properties and Processing Target Textile: A Review. Materials.

[5] Dai D., Fan M. (2010). Characteristic and Performance of Elementary Hemp Fibre. Mater. Sci. Appl..

[6] Di Mola I., Conti S., Cozzolino E., Melchionna G., Ottaiano L., Testa A., Sabatino L., Rouphael Y., Mori M. (2021). Plant-Based Protein Hydrolysate Improves Salinity Tolerance in Hemp: Agronomical and Physiological Aspects. Agronomy.

[7] Zimniewska M., Rozanska W., Gryszczynska A., Romanowska B., Kicinska-Jakubowska A. (2018). Antioxidant Potential of Hemp and Flax Fibers Depending on Their Chemical Composition. Molecules.

[8] Dorado J., Almendros G., Field J.A., Sierra-Alvarez R. (2001). Infrared spectroscopy analysis of hemp (*Cannabis sativa*) after selective delignification by *Bjerkandera* sp. at different nitrogen levels. Enzym. Microb. Technol..

[9] Zhang J., Gao J., Chen Y., Hao X., Jin X. (2017). Characterization, preparation, and reaction mechanism of hemp stem based activated carbon. Results Phys..

[10] Pacheco G., Véspoli de Mello C., Chiari-Andréo G., Borges Isaac V.L., Sidney José Lima Ribeiro S.J., Édison Pecoraro E., Trovatti E. (2018). Bacterial cellulose skin masks-Properties and sensory tests, Review. J. Cosmet. Dermatol..

[11] Tang J., He H., Wan R., Yang Q., Luo H., Li L., Xiong L. (2021). Cellulose Nanocrystals for Skin Barrier Protection by Preparing a Versatile Foundation Liquid. ACS Omega.

[12] Pilar Vinardell M., Montserrat Mitjans M. (2017). Lignins and Their Derivatives with Benefificial Effects on Human Health. Int. J. Mol. Sci..

[13] Shena Y., Songa X., Lic L., Sun J., Jaiswal Y., Huang J., Liu C., Yang W., Williams L., Zhang H. (2019). Protective effffects of p-coumaric acid against oxidant and hyperlipidemia-an in vitro and in vivo evaluation. Biomed. Pharmacother..

[14] Chool Boo Y. (2019). p-Coumaric Acid as An Active Ingredient in Cosmetics: A Review Focusing on its Antimelanogenic Effects. Antioxidants.

[15] Aldaba-Muruato L.R., Ventura-Juárez J., Perez-Hernandez A.M., Hernández-Morales A., Muñoz-Ortega M.H., Martínez-Hernández S.L., Alvarado-Sánchez B., Macías-Pérez J.R. (2021). Therapeutic perspectives of p-coumaric acid: Anti-necrotic, anti-cholestatic and anti-amoebic activities. World Acad. Sci. J..

[16] Zduńska K., Dana A., Kolodziejczak A., Rotsztejn H. (2018). Antioxidant Properties of Ferulic Acid and Its Possible Application. Ski. Pharmacol. Physiol..

[17] Li Y., Fu Q., Yu S., Min Yan M., Berglund L. (2016). Optically Transparent Wood from a Nanoporous Cellulosic Template: Combining Functional and Structural Performance. Biomacromolecules.

[18] Alipoormazandarani N., Fokkink R., Fateh P. (2021). Deposition behavior of lignin on solid surfaces assessed by stagnation point adsorption reflectometry. RSC Adv..

[19] Chen J., Liu C., Wu S., Liang J., Lei M. (2016). Enhancing the quality of bio-oil from catalytic pyrolysis of kraft black liquor lignin. RSC Adv..

[20] Puiu M., Bala C. (2022). Affifinity Assays for Cannabinoids Detection: Are They Amenable to On-Site Screening?. Biosensors.

[21] Cocean G., Cocean A., Postolachi C., Garofalide S., Bulai G., Munteanu B.S., Cimpoesu N., Cocean I., Gurlui S. (2022). High-Power Laser Deposition of Chitosan Polymers: Medical and Environmental Applications. Polymers.

[22] Thirumal S., Duraikannu G. (2019). Fourier-transform Infrared Analysis and In Vitro Antibacterial Activity of Ormocarpum cochinchinense (Elumbotti). Int. J. Pharm. Biol. Arch..

[23] Blanchard M., Meheut M., Delon L., Poirier M. (2018). Pierre Micoud, Christophe Le Roux, François Martin Infrared spectroscopic study of the synthetic Mg–Ni talc series. Phys. Chem. Miner..

[24] Pretsch E., Buhlmann P., Badertscher M. (2009). Structure Determination of Organic Compounds. Tables of Spectral Data.

[25] Hussien S.S. (2019). Biosynthesis, Extraction and Characterization of Extracellular Polymeric Substances (EPSs) from *Aspergillus clavatus*. Adv. Environ. Stud..

[26] Cocean A., Postolachi C., Cocean G., Bulai G., Munteanu B.S., Cimpoesu N., Cocean I., Gurlui S. (2022). The Origin and Physico-Chemical Properties of Some Unusual Earth Rock Fragments. Appl. Sci..

[27] Cocean A., Cocean I., Cimpoesu N., Cocean G., Cimpoesu R., Postolachi C., Popescu V., Gurlui S. (2021). Laser Induced Method to Produce Curcuminoid-Silanol Thin Films for Transdermal Patches Using Irradiation of Turmeric Target. Appl. Sci..

[28] Cocean A., Cocean I., Cocean G., Postolachi C., Pricop D.A., Munteanu B.S., Cimpoesu N., Gurlui S. (2021). Study of Physico-Chemical Interactions during the Production of Silver Citrate Nanocomposites with Hemp Fiber. Nanomaterials.

[29] Cocean I., Cocean A., Postolachi C., Pohoata V., Cimpoesu N., Bulai G., Iacomi F., Gurlui S. (2019). Alpha keratin amino acids behvior under high fluence laser interaction. Medical applications. Appl. Surf. Sci..

[30] De Pascale M., De Angelis M.G., Boi C. (2022). Mixed Matrix Membranes Adsorbers (MMMAs) for the Removal of Uremic Toxins from Dialysate. Membranes.

[31] Cocean A., Pelin V., Cazacu M.M., Cocean I., Sandu I., Gurlui S., Iacomi F. (2017). Thermal effects induced by laser ablation in non-homogeneous limestone covered by an impurity layer. Appl. Surf. Sci..

[32] Cocean A., Cocean I., Gurlui S., Iacomi F. (2017). Study of the pulsed laser deposition phenomena by means of Comsol Multiphysics. Univ. Politeh. Buchar. Sci. Bull. Ser. A Appl. Math. Phys..

[33] Cocean A., Cocean I., Gurlui S. (2021). Influence of the Impurities to the Composite Materials in Laser Ablation Phenomena. Univ. Politeh. Buchar. Sci. Bull. Ser. A Appl. Math. Phys..

[34] Kawamoto H. (2017). Lignin pyrolysis reactions. J. Wood Sci..

[35] Buschmann C., Langsdorf G., Lichtenthaler H.K. (2000). Imaging of the blue, green, and red fluotrescence emission of plants: An overview. Photosynthetica.

[36] Ndolo V.U., Beta T., Fulcher R.G. (2013). Ferulic acid fluorescence intensity profifiles and concentration measured by HPLC in pigmented and non-pigmented cereals. Food Res. Int..

[37] Garcia-Sanchez F., Carnero C., Heredia A. (1988). Fluorometric determination of p-coumaric acid in beer. J. Agric. Food Chem..

[38] Putschögl M., Zirak P., Penzkofer A. (2008). Absorption and emission behaviour of trans-p-coumaric acid in aqueous solutions and some organic solvents. Chem. Phys..

[39] Optical Constants of Ca (Calcium). https://refractiveindex.info/?shelf=main&book=Ca&page=Mathewson.

